# Microbe-induced gene silencing of fungal gene confers efficient resistance against *Fusarium graminearum* in maize

**DOI:** 10.1007/s42994-025-00212-9

**Published:** 2025-04-14

**Authors:** Ting Chen, Wen Tian, Qing Shuai, Han-Guang Wen, Hui-Shan Guo, Jian-Hua Zhao

**Affiliations:** 1 State Key Laboratory of Plant Genomics, Institute of Microbiology, Chinese Academy of Sciences, Beijing, 100101 China; 2 CAS Center for Excellence in Biotic Interactions, University of Chinese Academy of Sciences, Beijing, 100101 China

**Keywords:** RNAi, MIGS, *Fusarium graminearum*, Maize

## Abstract

Small RNAs (sRNAs), the main effectors of RNA interference (or RNA silencing, RNAi), mediate cell-autonomous and non-cell-autonomous gene silencing. The discoveries of trans-kingdom RNAi and interspecies RNAi have accelerated the development of RNAi-based crop protection technologies. Recently, based on interspecies RNAi, a practical technology termed microbe-induced gene silencing (MIGS) without the need of host genetic modification is developed for crop protection against *Verticillium dahliae* and *Fusarium oxysporum* in cotton and rice plants. In this study, we utilized MIGS technology to protect maize against *Fusarium graminearum*, which is responsible for maize stalk rot. An RNAi-engineered *Trichoderma harzianum *strain, Th-FgPmt2i, was exploited to generate double-stranded RNAs (dsRNAs) to trigger the silencing of the *FgPTM2* gene. Our data verify that sRNAs generated from Th-FgPmt2i can silence the *FgPMT2* gene via translational inhibition in *F. graminearum*. We further demonstrated that Th-FgPmt2i has a stronger capacity than does the *T. harzianum* chassis for protection of maize against *F. graminearum*. Coupled with our studies on crop protection against *V. dahliae* and *F. oxysporum*, our findings reveal that MIGS can be exploited to protect various crops against distinct fungal pathogens and has extensive applicability.

**Supplementary Information:**

The online version contains supplementary material available at 10.1007/s42994-025-00212-9.

Dear Editor,

Stalk rot, which is frequently caused by a mixed invasion of multiple causal pathogens, is a devastating disease in maize worldwide. *Fusarium graminearum* has the highest pathogenicity and aggressiveness among the pathogens responsible for maize stalk rot (Cao et al. 2023; Zhang et al. 2016b). The lack of resistance genes and time consumption hinder progress toward maize breeding for controlling stalk rot (Yang et al. 2017).

In most eukaryotic organisms, double-stranded RNA (dsRNA)-triggered RNA interference (or RNA silencing, RNAi) is a conserved mechanism that regulates gene expression in a sequence-specific manner (Xie and Duan 2023; Zhao and Guo 2022). As key factors of RNAi, small RNAs (sRNAs) not only mediate cell-autonomous gene silencing but also act in a noncell-autonomous manner (Zhao and Guo 2022; Li et al. 2024). Owing to their target specificity and easy design, RNAi-based strategies have been successfully applied to protect crops against various pathogens (Guo et al. 2019; Hou and Ma 2020; Wang and Jin 2017; Zhao et al. 2021, 2024). Host-induced gene silencing (HIGS) is an ideal strategy for disease resistance breeding because it is independent of resistant cultivars, effective and environmentally friendly, in which dsRNAs are expressed in plants to silence pathogenic genes (Hua et al. 2018; Nowara et al. 2010). The discovery of natural trans-kingdom RNAi highlights the promise of HIGS for crop protection (Zhang et al. 2016a). However, a lack of transformation technology in many crop species limits the broader application of HIGS (Zhao et al. 2024).

Recently, we developed an interspecies RNAi-based strategy termed microbe-induced gene silencing (MIGS) to control fungal diseases, where *Trichoderma harzianum,* as a chassis, produces dsRNA to silence the *PMT2* gene (encoding the *O*-mannosyltransferase) in two soil-borne pathogenic fungi, *Verticillium dahliae* and *Fusarium oxysporum*. Compared with the chassis, the engineered *T. harzianum* strains presented a greater capacity for cotton and rice protection against *V. dahliae* and *F. oxysporum* (Wen et al. 2023). The MIGS strategy does not rely on the collection of resistant cultivars and avoids crop genetic modifications; thus, as an environmentally friendly method for improving agriculture, the use of traditional chemical compounds in field crops may be reduced (Fang 2024).

To identify the roles of *F. graminearum PMT2* in the fungal development and virulence, we cloned the *FgPMT2* gene and attempted to knockout the *FgPMT2* gene by homologous recombinants as previously described (Fig. S1A) (Wang et al. 2016). We failed to obtain the *FgPMT2* deletion strain, indicating that FgPMT2 is essential for *F. graminearum*. Therefore, two *FgPMT2*-specific fragments were selected to generate an RNAi construct (FgPmt2i) for *T. harziamum* transformation, resulting in Th-FgPmt2i (Fig. S1B, C). Similar colony morphologies and growth rates were observed between transformants and wild-type *T. harziamum* (Th) (Fig. S1D). FgPmt2i-derived sRNAs (siPmt2s) were examined via RNA gel blotting. Various accumulation levels were detected in different Th-FgPmt2i strains (Fig. 1A). Th-FgPmt2i-1, which produced the most sRNAs, was used in the following study.

**Fig. 1:**
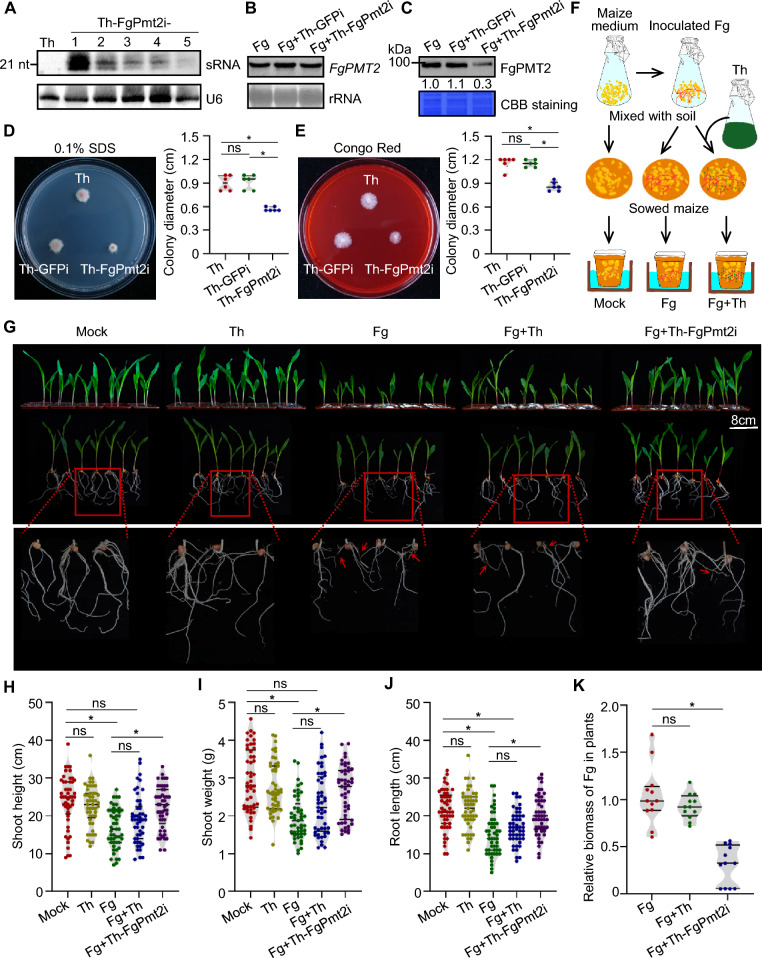
MIGS for protection of maize against *F. graminearum* by silencing the *FgPMT2* gene. **A** Detection of Th-FgPmt2i-generated siPmt2s via RNA gel blotting. U6 was detected as a loading control. **B** *FgPMT2* mRNA was examined by RNA gel blotting with a specific probe. Ribosomal RNA (rRNA) was used as a loading control. **C** Detection of FgPMT2 protein accumulation with an anti-FgPMT2 antibody. Coomassie brilliant blue (CBB) staining served as a loading control. **D, E** Susceptibility of *F. graminearum* to 0.1% SDS (**D**) and 300 µg/ml Congo red (**E**). *F. graminearum* incubated with culture supernatants of various Th strains were grown on PDA plates. Violin plots showing the fungal colony diameters. Each point represents an independent replicate (*n* = 6). **F** Diagram of the maize protection assay. **G** Effects of Th-FgPmt2i on maize seedling protection against *F. graminearum*. The symptoms were recorded at 10 days post sowing (dps). The arrow indicates brown lesions in the roots. **H-J** The shoot height (H), shoot weight (I) and root length (J) of maize in each indicated inoculum at 10 dps. Each point represents an independent plant (*n* = 49). **K** The relative biomass of Fg in infected maize seedlings at 10 dps. Each point represents an independent replicate (*n* = 12). The medians and quartiles are indicated by red and black lines, respectively. The asterisk indicates a significant difference according to the Kruskal–Wallis test (*P* < 0.05) (**D**, **E**, **H**–**K**)

The RNA uptake ability of fungal pathogens is crucial for the development of successful MIGS, so we first examined whether *F. graminearum* can take up RNA from the environment. The fluorescently labeled homologous dsRNAs of *FgPMT2* were incubated with *F. graminearum*. After culturing for 36 h, fluorescence signals were clearly observed within the hyphae after micrococcal nuclease (MNase) treatment, indicating that exogenous dsRNAs can be absorbed by *F. graminearum* (Fig. S2). To test whether the siPmt2s generated in Th-FgPmt2i were able to mediate *FgPMT2* silencing in *F. graminearum*, we incubated *F. graminearum* with the culture supernatants of Th-FgPmt2i strains. The supernatants of the wild-type (WT) Th and Th-GFPi strains generated in our previous study (Wen et al. 2023) were used as controls. Similar *FgPMT2* mRNA accumulation was detected in *F. graminearum* (Fg) incubated with or without supernatants from Th strains (Fig. 1B and Fig. S3). Western blot analysis revealed that the FgPMT2 protein levels were significantly lower in Fg incubated with supernatants of Th-FgPmt2i than in those incubated with supernatants of WT Th or Th-GFPi (Fig. 1C). These results demonstrate that the siPmt2s generated in Th-FgPmt2i could silence the *FgPMT2* gene via translational inhibition in Fg. A previous study revealed that deletion or suppression of *PMT* genes damages fungal cell wall integrity, increasing hypersensitivity to antifungal reagents (Xu et al. 2020). We therefore examined whether siPmt2-mediated FgPMT2 translation inhibition could damage the cell wall integrity of *F. graminearum*. Fg strains treated with supernatants of Th strains were inoculated onto PDA media supplemented with SDS or Congo red. Compared with incubation with Th or Th-GFPi supernatants, incubation with Th-FgPmt2i supernatants significantly reduced the diameter of fungal colonies, indicating that the siPmt2s released into the supernatants increased the sensitivity of *F. graminearum* to SDS and Congo red (Fig 1D and E). Together, our data indicated that Fg cell wall integrity was damaged by siPmt2-induced FgPMT2 suppression.

Next, we tested whether Th-FgPmt2i had beneficial plant protection effects against *F. graminearum* infection. The maize seeds were sown in soil containing the Fg or Fg + Th strains (Fig. 1F). Maize seedlings grown in soil (mock) or Th-soil as controls presented similar growth rates. The shoot height and weight, as well as the root length, of the maize seedlings did not obviously differ (Fig 1G–J). In the Fg-soil, growth retardation of the seedlings and brown lesions were detected (Fig 1 G–J). Similarly, growth retardation was detected for the seedlings in the Fg + Th-soil, with somewhat fewer brown lesions in the roots observed, suggesting that WT Th has a certain effect on the protection of maize seedlings against Fg. Compared with those in the Fg + Th-soil, most maize seedlings developed well in the Fg + Th-FgPmt2i-soil. The growth phenotypes of the seedlings in the Fg + Th-FgPmt2i-soil were significantly greater than those in the Fg-soil treatment (Fig. 1G-J). Only a few brown lesions were observed in the roots of the plants in the Fg + Th-FgPmt2i-soil (Fig. 1G). The disease severity index (DSI) was significantly lower for seedlings in the Fg + Th-FgPmt2i-soil than those in the Fg-soil and Fg + Th-soil (Fig. S4). Consistent with the growth phenotypes, the relative Fg biomass in roots in the Fg + Th-FgPmt2i-soil significantly decreased compared with that in the Fg-soil and Fg + Th-soil (Fig. 1K). The development of Fg hyphae reaching the soil surface on the pots (white color) was observed, and it was markedly lower in the Fg + Th-FgPmt2i-soil than in the Fg-soil and Fg + Th-soil (Fig. 1G). Together, our data showed that engineered Th-Pmt2i had a stronger capacity than Th for maize protection against *F. graminearum*.

Disease resistance breeding is the main chemical pesticide-free strategy for crop improvement. However, cross-breeding and transgenic breeding are restricted by a lack of resistance resources (Zhao et al. 2024). Recently, we revealed the existence of interspecies RNAi in fungi and established a MIGS system by using engineered *T. harzianum*. In this study, we successfully established a MIGS system to protect maize against *F. graminearum* by targeting the *FgPMT2* gene. Our results demonstrated that MIGS is an extensively applicable strategy for crop protection against fungal pathogens.

## Materials and methods

### *F. graminearum* inoculation

The fungal pathogen *F. graminearum* was a gift from Yan-Tao Jia (Institute of Microbiology, Chinese Academy of Sciences). *F. graminearum* was first inoculated into the corn culture medium at 26 °C for 10 days when mycelium covered the maize seeds. For the fungal infection assays, the infected maize was mixed with the soil (1:3, v/v). Maize seeds (zhengdan 958) were sown into premixed soil. Soil mixed with *T. harzianum* strains was used to examine the protective effect of engineered *T. harzianum*. The spores of each *T. harzianum* strain were mixed into the soil at a final concentration of 10^6^ c.f.u.g^−1^. The maize seedlings were grown in a glasshouse at 25 °C with a 16 h light/8 h dark cycle (Fig. 1F). At 10 dps, photographs were recorded, and the seedlings were collected. Disease severity was scored at 10 dps with a rating scale of 1–5 according to previous studies (Cao et al. 2023; Ye et al. 2019). DSI (%) = ∑(grade × number of plants in grade) × 100/(5 × total number of plants).

The *F. graminearum* biomass in infected seedlings was estimated by the ratio of fungal-specific DNA to maize-specific DNA. The primers used are listed in Table S1.

### dsRNA design and fungal transformation

According to the reference sequence (FGSG_01535), we cloned the FgPMT2 sequence with specific primers (Table S1) and selected two fragments to ligate to the intron of *VdTUBULIN* to generate the RNAi construct FgPmt2i (Fig. S1B). The selected fragments for the RNAi construct were synthesized by GenScript and ligated to the *Pac* I/*Bam*H I-linearized pNeo plasmid.

To generate Th-FgPmt2i strains, fungal transformation was performed as previously described (Wen et al. 2023). The Th-GFPi strain was described in our previous study (Wen et al. 2023).

### Fungal uptake of fluorescein-labeled dsRNA in vitro

In vitro synthesis of fluorescein-labeled dsRNA (shown in Fig. S1B) was performed with a T7 RNAi Transcription Kit (Vazyme, TR102-01). Fluorescein RNA Labeling Mix (Roche, 11,685,619,910) was used to label dsRNA following the manufacturer’s instructions.

To examine dsRNA uptake by *F. graminearum*, dsRNAs were drooped onto a cellophane membrane with mycelium. After incubation for 36 h, MNase treatment was performed to remove the dsRNAs outside the fungal cells. The fluorescent signal was analyzed via a Leica SP8 confocal microscope.

### DNA extraction and southern blot analysis

Genomic DNA was extracted from *T. harzianum* strains and maize roots via the CTAB method (Allen et al. 2006). For Southern blot analysis, 20 µg of total DNA was digested overnight with *Bam*H I. The enzyme-digested products were separated on a 0.9% agarose gel and transferred to a nylon membrane (Amersham Hybond-N^+^ membrane, GE, RPN303B). Biotin-11-dUTP (Thermo Fisher, R0081)-labeled probes were added to the hybridization mixture (Sigma, H7033-1 L) and hybridized overnight at 65 °C. A Chemiluminescent Nucleic Acid Detection Module kit (Thermo Fisher, 89,880) and a fully automatic chemiluminescence image analysis system (Tanon, Tanon-4600SF) were used to detect the hybridization signals and obtain images, respectively. The primers used are listed in Table S1.

### Fungal cell wall integrity analysis

To examine the cell wall integrity of *F. graminearum* with *FgPMT2* gene suppression, *F. graminearum* and *T. harzianum* strains were cultured in CM and Czapek-dox liquid media, respectively, at 28 °C for 48 h. The mycelia of *T. harzianum* were separated and discarded via centrifugation (Eppendorf Centrifuge 5810R) at 2755×*g* for 10 min. 0.22 µm filters (Millex 33 mm PES, SLGPR33R) were used to remove the residual cells from the supernatants. *F. graminearum* was mixed with the supernatants of *T. harzianum* (1:1, v/v) and cultured at 28 °C. After 24 h, *F. graminearum* was inoculated on PDA plates containing Congo Red (300 µg/mL) or 0.1% SDS, and *T. harzianum* supernatants were added every 8 h. After 24 h, photographs were recorded.

### RNA extraction, RNA gel blotting and RT-qPCR

A Total RNA Isolation Kit Plus (Foregene, RE-05021) was used to extract total RNA. For mRNA detection, 15 µg of total RNA was separated on a 1.2% agarose gel and transferred to a nylon membrane (Amersham Hybond-N + membrane, GE, RPN303B). The hybridization and signal detection followed the procedures of Southern blot analysis.

For sRNA detection, 20–30 µg of total RNA was separated on a 17% denaturing polyacrylamide gel and transferred to a nylon membrane (Amersham Hybond-N + membrane, GE, RPN303B). The probes were synthesized by RuiBiotech and labeled with biotin at the 3’ end. For hybridization, the mylon membrane was incubated with biotin-labeled probes overnight at 42 °C. Hybridization detection followed the procedures of Southern blot analysis.

RT-qPCR was performed following the procedure of the ChamQ Universal SYBR qPCR Master Mix (Vazyme, Q711-02). The CFX96 Touch Real-Time PCR detection system was used to analyze the results. Three biological replicates and three technical replicates were performed and are shown in Fig. S3. The sequences of the primers and probes used are listed in Table S1.

### Protein extraction and western blot analysis

The buffer (10 mM Tris (pH 8.0), 150 mM NaCl, 0.5 mM EDTA, 1% Triton X-100, and 1 × protease inhibitor) was used to extract the total protein. Total proteins were separated via polyacrylamide gel electrophoresis and transferred onto PVDF membranes (Merck Millipore, IPVH00010). The membranes were blotted with primary anti-FgPMT2 (GenScript) and horseradish peroxidase-conjugated goat anti-rabbit antibodies (Easybio, BE0123-100) at a dilution of 1:5,000. A fully automatic chemiluminescence image analysis system (Tanon, Tanon-4600SF) was used to detect the bands.

## Supplementary Information

Below is the link to the electronic supplementary material.

Supplementary file1 (PDF 573 KB)

## Data Availability

All the data for this study are presented in the paper or the supplementary materials.
