# Supplementary material for: Microbe-induced gene silencing of fungal gene confers efficient resistance against Fusarium graminearum in maize
Source: aBIOTECH. 2025 Apr 14;6(3):466–71. doi: 10.1007/s42994-025-00212-9 (PMC12454818; doi:10.1007/s42994-025-00212-9)
Supplement: Supplementary file 1 — Supplementary file1 (PDF 573 KB) [file 42994_2025_212_MOESM1_ESM.pdf]

Supplementary Information

Microbe-induced gene silencing of fungal gene confers efficient resistance against *Fusarium graminearum* in maize

Ting Chen[1,2], Wen Tian[1,2], Qing Shuai[1,2], Han-Guang Wen[1,2], Hui-Shan Guo[1,2], Jian-Hua Zhao[1,2*]

[1] State Key Laboratory of Plant Genomics, Institute of Microbiology, Chinese Academy of Sciences, Beijing 100101, China.

[2] CAS Center for Excellence in Biotic Interactions, University of Chinese Academy of Sciences, Beijing 100101, China.

[*] Correspondence: zhao_jian_hua@hotmail.com (J.H.Z)

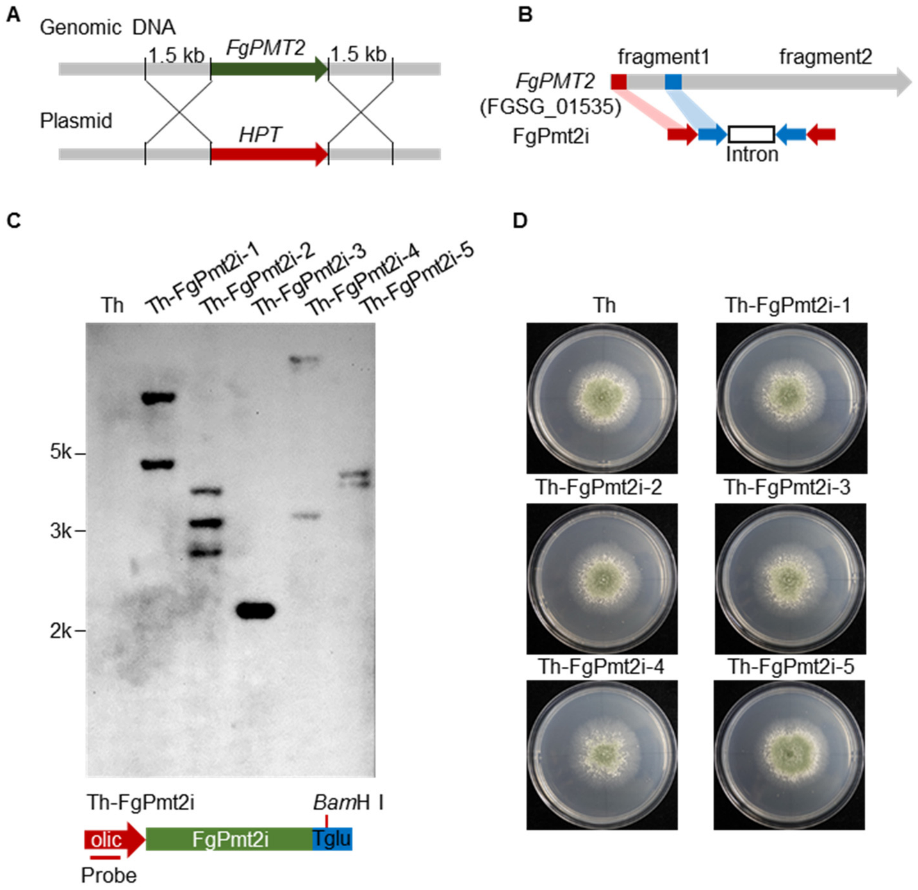

Fig. S1 Detection of Th-FgPmt2i transformants. **A** Schematic description of the homologous recombination event involving *FgPMT2* deletion. **B** Schematic description of the generation of FgPMT2i. **C** Detection of Th-FgPmt2i transformants by southern blotting. The restriction enzyme and probe are indicated. **D** Th-FgPmt2i strains showed normal colony morphology compared with the Th strain

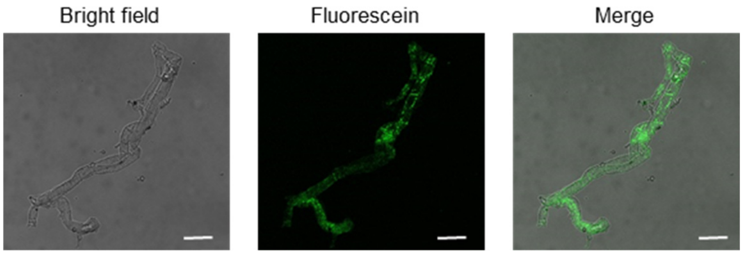

Fig. S2 Examination of dsRNA uptake in *F. graminearum*. Fluorescein-labeled dsRNA was drooped on the hyphae. Bar = 10 μm

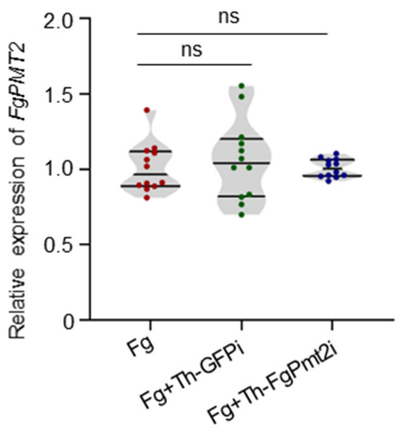

Fig. S3 Detection of *FgPMT2* expression by RT-qPCR. Each point represents an independent replicate (n=12). Medians and quartiles are indicated by red and black lines, respectively. 'ns' indicates no significant difference according to the Kruskal–Wallis test ($P > 0.05$)

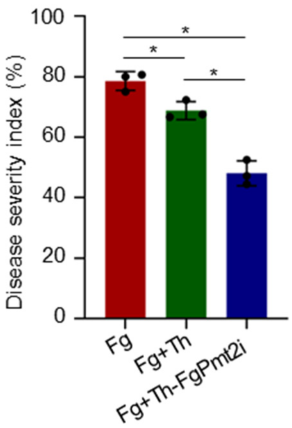

Fig. S4 Disease severity index at 10 dps for seedlings in the Fg-, Fg+Th- and Fg+Th-FgPmt2i-soil. Each point represents an independent replicate. The data are presented as the means ± SD. '*' indicates a significant difference according to the one-way ANOVA with Tukey's multiple comparisons test ($P < 0.05$)

Table S1 Primers used in this study

Name	Primer Sequence(5'-3')	Purpose
siPmt2s	ATGGCCGCCGCCGACAAGGCT<br>TGCTGGACAAGCCAACCTGCA<br>AACAAGAAGCTCATAAAGAAG<br>GAGCCTTCGTTCCTCGATACC<br>CCCCGCTGATCTTTACACTCC<br>TGTGACATGGGATGAGGCTCA	Northern Blotting
Ref_F	TCCGTAGGTGAACCTGCGG	RT-qPCR and cloned *FgPMT2*. FgPMT2_F/R_c were used for cloning
Ref_R	TCCTCCGCTTATTGATATGC	
FgPMT2_F/<br>FgPMT2_F_c	ATGGCCGCCGCCGACAA	
FgPMT2_R	TGAGCCTCATCCCATGT	
FgPMT2_R_c	TTAGTCGGTGACTCGCCAGTTG	
GAPDH_F	CCATCACTGCCACACAGAAAAC	Biomass detection
GAPDH_R	AGGAACACGGAAGGACATACCAG	
Fg_F	CTCCGGATATGTTGCGTCAA	
Fg_R	GGTAGGTATCCGACATGGCAA	
Olic_F	AGACGTATTTAGGTGCTAGGGC	Sorthern blotting
Olic_R	CTCCACAAGGGTCCATGCTA	
